# Autophagy—A Hidden but Important Actor on Oral Cancer Scene

**DOI:** 10.3390/ijms21239325

**Published:** 2020-12-07

**Authors:** Totan Alexandra, Imre Melescanu Marina, Miricescu Daniela, Stanescu Iulia Ioana, BencZe Maria, Radulescu Radu, Tancu Ana Maria, Spinu Tudor, Greabu Maria

**Affiliations:** 1 Department of Biochemistry, Faculty of Dental Medicine, University of Medicine and Pharmacy Carol Davila, 8 Eroilor Sanitari Blvd, 050474 Bucharest, Romania; alexandra.totan@umfcd.ro (T.A.); daniela.miricescu@umfcd.ro (M.D.); iulia.stanescu@umfcd.ro (S.I.I.); radu.radulescu@umfcd.ro (R.R.); maria.greabu@umfcd.ro (G.M.); 2 Department of Complete Denture, Faculty of Dental Medicine, University of Medicine and Pharmacy Carol Davila, 17-23 Calea Plevnei, 010221 Bucharest, Romania; anamaria.tancu@umfcd.ro; 3 Department of Orthodontics and Dental-Facial Orthopaedics, Faculty of Dental Medicine, University of Medicine and Pharmacy Carol Davila, 17-23 Calea Plevnei, 010221 Bucharest, Romania; 4 Department of Fixed Prosthodontics and Occlusion, Faculty of Dental Medicine, University of Medicine and Pharmacy Carol Davila, 17-23 Calea Plevnei, 010221 Bucharest, Romania; tudor.spinu@umfcd.ro

**Keywords:** oral cancer, autophagy, PI3K/AKT/mTOR signaling pathway

## Abstract

The duration of denture use, oral hygiene, smoking and male sex were identified as risk factors for oral mucosal lesions. As it is well known, all the oral mucosal lesions associated with risk factors have an important degree of malignity. Chronic mechanical irritation can be another cause of oral cancer and it is produced by the constant action of a deleterious agent from the oral cavity. Autophagy represents a complex evolutionary conserved catabolic process in which cells self-digest intracellular organelles in order to regulate their normal turnover and remove the damaged ones with compromised function to further maintain homeostasis. Autophagy is modulated by mTOR kinase and indirectly by PI3K/AKT survival pathway. Due to its dual capacity to either induce cell death or promote cell survival, important evidence pointed that autophagy has a two-faced role in response to chemotherapy in cancer. In conclusion, understanding how to overcome cytoprotective autophagy and how to take advantage of autophagic cell death is critical in order to enhance the cancer cells sensitivity to particular therapeutic agents.

## 1. Introduction

Oral mucosal lesions usually occur due to systemic diseases, nutritional disorders, medication side effects or wearing ill-fitting dentures in the elderly [1]. The most common oral mucosal lesions in the aging population can be caused by both poor oral hygiene and continuous use of dentures throughout the day and night [2]. Apart from the duration of denture use, smoking and male sex were also identified as risk factors for oral mucosal lesions, with fissured tongue and lingual varicosity being the most common forms [3,4].

As it is well known, all the oral mucosal lesions associated with risk factors have an important degree of malignity. The oral cavity is one of the most appropriate locations for the development of oncological diseases, especially in patients who are >40 years old. Malignant lesions were determined in only men in the study by Dundar and Ilhan Kal and in only women in a study by Cebeci et al. [5].

Autophagy represents a complex evolutionary conserved catabolic process in which cells self-digest intracellular organelles in order to regulate their normal turnover and remove the damaged ones with compromised function, to further maintain homeostasis [6,7,8,9].

Currently, the role of autophagy in cancer is still controversial. On the one hand, constitutive autophagy can be regarded as a cellular housekeeper that eliminates damaged organelles and protect cells against carcinogenesis, and moreover it has been shown that excess or persistent autophagy promotes cell death by inducing apoptosis or mediating “autophagic cell death”. However, on the other hand, it can also act as a pro-survival signal in response to stress (like nutrient deprivation, hypoxia and the presence of chemotherapy or some other targeted therapies) that could induce resistance to anticancer therapies in advanced cancer [7,8,9].

Autophagy can control many cellular molecular pathways involved in tumor promotion and suppression, immune response intensity. A lot of studies have focused on its involvement in these processes as a modulator of pathogenesis and, consequently, as a potential therapeutic target.

In this paper, we review recent progress and provocation in our understanding of how to overcome cytoprotective autophagy and how to take advantage of autophagic cell death in order to enhance cancer cells’ sensitivity to particular therapeutic agents.

## 2. Oral Cancer

Head and neck region cancers are one of the most common types of cancers, oral cancer being the sixth most common malignancy in the world, and is characterized by a very low five-year survival rate, about 50% due to late stage diagnosis, high degree of invasiveness and development of therapeutic resistance [10,11,12,13]. Almost all of the oral cancers (90%) are squamous cell carcinomas with various levels of cell differentiation and lymph nodes metastasis [10,13,14,15,16]. The other 10% of oral cancers originate from connective tissue, minor salivary glands, lymphoid tissue or melanocytes malignant processes [11,17].

According to the latest reports of the International Agency for Research on Cancer (IARC) for oral cancer (ICD-10 code C00-08: Lip, Oral Cavity), the annual incidence is higher over 300.000 diagnosed cases, and the annual mortality is about 145,000 death [18]. The regions characterized by a high incidence of oral cancer are found in South and Southeast Asia (Sri Lanka, India, Pakistan and Taiwan), areas of the West (France) and Eastern Europe (Hungary, Slovakia and Slovenia), Latin America and the Caribbean (Brazil, Uruguay and Puerto Rico) and Pacific regions (Papua New Guinea and Melanesia) [10,18].

Oral cancers can be located anywhere in the oral region that extends anatomically from the lip vermilion to the soft and hard palate junction and the circumvallate papillae of the tongue [18].

Oral cancer can be caused by genetic factors, epigenetic modifications (such as histones modifications; nucleosome integrity, DNA methylation and expression of non-coding RNAs (ncRNAs), tobacco and alcohol consumption, chronic infections such as human papilloma virus (HPV) or syphilis infections, dental factors, occupational risks [13,19].

Tobacco consumption is the main risk factor in oral cancer development and is responsible for other types of cancer also, such as lung, pharynx, larynx, esophagus, urinary bladder, renal, pelvis, and pancreas cancers [20,21]. The use of tobacco increases the risk of developing oral cancer by three times compared to non-smokers [22]. The main carcinogenetic factors found in tobacco smoke are nitrosamines, benzopyrenes and aromatic amines that undergo various enzymatic and non-enzymatic transformations resulting in molecules that are covalent bound to various regions of DNA resulting in DNA adducts and various mutations [22]. Tobacco consumption also generates a high oxidative stress via the high concentration of free radicals contained, both oxygen and nitrogen species, that deplete enzymatic and non-enzymatic cellular antioxidants resulting in cell damage leading to cancer [23].

Alcohol consumption can act as a local or systemic risk factor in oral cancer development. Systemic effects of alcohol consumption are related to the accumulation of acetaldehyde, the main metabolite of ethanol, that causes genetic alterations through disruption of DNA synthesis and repair mechanisms [19,24]. Acetaldehyde can also be produced by oral bacteria [1]. Locally, alcohol can increase the permeability of the oral mucosa for other carcinogenetic factors such as those found in tobacco and thus, working synergistically with tobacco carcinogens [16]. Alcohol can also induce epithelial atrophy, further increasing oral mucosa permeability, it’s effects being directly linked with the intensity and duration of the chronic consumption [25].

In the oral cavity the human papilloma virus can be found near undifferentiated basal keratinocytes and mainly in the tonsillary crypts and the base of the tongue [16,26]. The two types of HPV involved in oral cancer development are HPV 16 and HPV 18 and they act by blocking or altering the expression of essential nuclear proteins such as 53, P21 and P16, thus inducing the transformation of normal cells into malignant cells [27].

Chronic mechanical irritation can be another cause of oral cancer and it is produced by the constant action of a deleterious agent from the oral cavity. The deleterious agents can be sharp and broken tooth, defective restorations, ill-fitting dentures with sharp or retentive edges or just constant biting of the oral mucosa that can sustain a chronic state of inflammation that induces epigenetic transformation of oral cells [28,29].

Oral cancer can evolve from a series of premalignant lesions, the most frequent being leukoplakia, submucosal fibrosis and erythroplakia [17]. Other less frequent premalignant lesions are oral lichen planus, actinic cheilitis, xeroderma pigmentosum and Fanconi’s anemia [30]. Leukoplakia is the most frequent of the premalignant lesions, affecting any part of oral cavity. It has several clinical forms such as homogeneous and non-homogeneous lesions and verrucous leukoplakia, the rate of malignant transformation depending on the localization of the lesion, the size and the duration and is around 1% [17,30]. Erythroplakia has a lower incidence compared to leukoplakia, but a higher rate of malignancy, between 14–50% due to high levels of dysplasia that accompany these lesions [30].

All regions of the oral cavity can develop malignant processes, but the most frequently affected sites are the tongue and the floor of the mouth, followed by the lip or the alveolar process, and are closely related to risk factors prevalence and lifestyle conditions usually following the “field cancerization concept” [31,32]. Tongue cancers usually develop in elderly patients, chronic exposure to alcohol and tobacco being the most frequent causes, this type of cancer being more aggressive with high relapsing and high invasiveness [33]. Cancers are caused mainly by environmental factors such as solar radiation, followed by smoking and viral infectious factors, and are overwhelmingly located at the lower lip (90%). Early detection and treatment ensure a very high five years survival rate (almost 80%) with few functional and aesthetical complications [34].

## 3. Autophagy

Autophagy is a survival-promoting pathway that captures, degrades, and recycles intracellular proteins and organelles in lysosomes. Autophagy preserves organelle function, prevents the toxic buildup of cellular waste products, and provides substrates to sustain metabolism in starvation. Although in some context autophagy suppresses tumorigenesis, in most contexts autophagy facilitates tumorigenesis. Cancers can upregulate autophagy to survive microenvironmental stress and to increase growth and aggressiveness. Mechanisms by which autophagy promotes cancer include suppressing induction of the P53 tumor suppressor protein and maintaining metabolic function of mitochondria. Efforts to inhibit autophagy to improve cancer therapy have thereby attracted great interest.

There are 3 primary forms of autophagy: macroautophagy, microautophagy and chaperone-mediated autophagy (CMA). The main differences between them concern their patterns of delivery and physiological functions [35]. Macroautophagy (referred to here after as autophagy) involves the formation of multiple membrane structures starting from the phagophore to autophagosome and, finally, to the autolysosome [35]. Autophagosome’s formation and consumption go through 4 steps: (1) induction and cargo packaging, (2) elongation of the phagophore, (3) autophagosome formation and completion, and (4) lysosomal fusion and breakdown [35]. The complex molecular process of autophagy is primarily dependent on the ATG (autophagy-related) family proteins [36].

Briefly, the molecular events sequence in autophagy is as follows:

(1) signals such as starvation activate the ULK complex, which will bind to the PtdIns3K complex following AMPK activation or mTOR suppression [35];
(2) following induction, the ULK complex, PtdIns3K complex and the ATG9 complex orchestrated action will trigger the phagophore assembly at the phagophore assembly site [35];
(3) ATG12 and LC3 conjugation systems are key players in regulating the phagophore elongation to the autophagosome. mTOR, the major autophagy inhibitory factor, suppresses autophagy as response to abundant nutrients conditions. This suppressive action is mediated by class I PI3K and AKT signaling [35];
(4) SQSTM1/p62 (sequestosome 1) receptor protein will consequently interact with both LC3 and ubiquitin chains [35];
(5) Further, the autophagosome will fuse with a lysosome, resulting the autolysosome formation. Inside autolysosome, the autophagosome constituents will be hydrolytically degraded. The trapped SQSTM1 will be degraded in the autolysosome, which highlight SQSTM1′s role as an autophagy flux marker [35].

### 3.1. Autophagy—An Important AKT/mTOR Pathway Target

AKT or protein kinase B was discovered in 1987 by Stephan Staal as the v-AKT- transforming gene component of the AKT-8 provirus. Eight years later, Richard Roth and his co-workers discovered that this kinase is activated by insulin [37,38]. AKT/PKB are serine/threonine kinases belonging to the kinase superfamily together with cAMP-dependent protein kinases (c-AMP), protein kinase A (PKA), protein kinase G (PKG) and protein kinase C (PKC), presenting structural homology within the catalytic domain and similar mechanisms of action [39].

**Figure 1:**
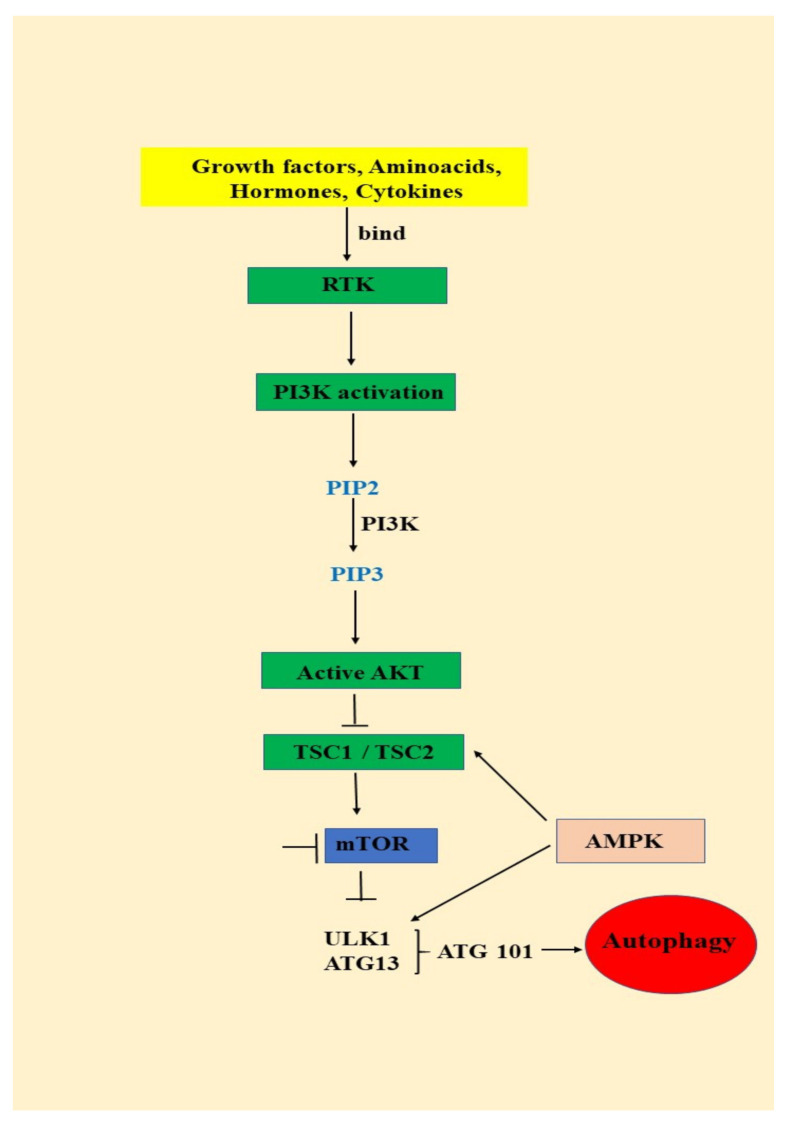
**The relationship between the PI3K/AKT/mTOR signaling pathway and autophagy: RTK** (receptor tyrosine kinase); **PI3K** (phosphatidylinositol 3-kinase); **PTEN** (phosphate and tensin homology); **AKT** (serine/threonine kinase); **TSC** (tuberous sclerosis complex); **mTOR** (mammalian target of rapamycin); **AMPK** (AMP-activated protein kinase); **ULK** (unc-51 like autophagy activating kinase ½); **ATG** (autophagy-related protein 13); **ATG 101** (autophagy-related protein 101).

AKT/mTOR signaling pathway is activated by growth factors and cytokines binding to the insulin receptor, which will lead to the activation of phosphatidylinositol 3-kinase (PI3K) and phosphorylation of phosphatidylinositol 3,4 bisphosphate (PIP2) to phosphatidylinositol 3,4,5 trisphosphate (PIP3) (Figure 1) PIP3 is an important secondary messenger that will determine the localization of AKT in the plasma membrane and is further phosphorylated by phosphoinositide dependent protein kinase-1 at the threonine 308. AKT maximum activation is achieved by the second phosphorylation that takes place at the Serine 473 by mTORC2 (mammalian target of rapamycin complex-2) (Figure 1) [39,40].

mTOR is a serine/threonine multicomponent kinase complex consisting of mTOR complex1 (mTOR1) and mTOR complex 2 (mTOR2). After activation, AKT phosphorylates TSC 1 (tuberous sclerosis complex 1) and TSC 2 (tuberous sclerosis complex 2) and inhibits them leading to mTOR1 activation. mTOR1 further phosphorylates 40S ribosomal protein kinase S6 (S6K) and eukaryotic initiating factor 4E binding protein (4EP1) and stimulates protein synthesis, metabolism and cell growth. Subsequently, activated AKT phosphorylates a series of proteins that are involved in glucose metabolism, cell proliferation and survival, but in the apoptosis process as well (Figure 1) [40].

PTEN is a negative regulator of AKT signaling pathway, being involved in the dephosphorylation of PIP3 to PIP2. Overactivation of AKT due to PTEN loss mediates the tumorigenesis process by tumor growth, survival and proliferation [41,42]. Cytokines, angiogenic and growth factors bind to the insulin receptor and activate AKT pathway. Unfortunately, overactivation of AKT signaling pathways is correlated with poor outcome for breast, prostate, endometrial, pancreatic, brain, gastric and melanoma cancers [36]. Moreover, this signaling pathway has been found to be, also, overactivated in oral cancer (Figure 1) [41,42,43,44,45,46].

Roy NK et al. also identified AKT isoforms specific to oral cancer, immunohistochemical analyzes reporting overexpression for AKT1 and AKT 2, but not for AKT3. In the case of head and neck cancers, genetic changes of AKT 1 and 2 are associated with a low survival rate. AKT1 and 2 isoforms are expressed in different regions of the oral cavity such as the tongue, cheek, and gingiva [47,48,49].

### 3.2. Autophagy—Important Actor on Oral Cancers Scene

Not surprisingly, the most molecular mechanisms involved in the autophagy regulation are deeply involved within signaling pathways with important roles in cancer control. Autophagy should be regarded as a molecular double-faced Janus God [50,51,52]. Thus, the tumor suppressors that negatively regulate mTOR (PTEN, AMPK, LKB1, and TSC1/2) will initiate autophagy machinery while, on contrary, oncogenes that activate mTOR (class I PI3K, Ras, and AKT), will inhibit autophagy. The role played by autophagy on the cancer scene depends on the genetic context, microenvironment, tumor type and stage of development [53].

Recent studies have illustrated in oral squamous cell carcinoma (OSCC) tissues or cell lines the existence of aberrant specific ATG protein expression profiles, such asATG9A, ATG5, ATG16L1, LC3 and BECN [53,54,55,56]. Experimental results have highlighted interesting correlations between various autophagy genes/proteins expression and OSCC prognosis, opening a challenging way to new biomarkers [51,54,57,58,59,60,61]. ATG9A is a transmembrane protein that regulates membrane delivery during autophagy pathway’s initial steps [62]. ATG9A overexpression has been showed to have a significant negative correlation with overall survival in OSCC patients. Consequently, ATG9A presence in the tumor cells cytoplasm should be regarded as a new candidate biomarker for the OSCC recurrence and survival [54,61].

ATG16L1 is also an essential actor in autophagosome formation. Experimental results highlighted its correlation with the unfavorable prognosis of patients with OSCCs. Elevated levels of ATG16L1 expression were detected in keratinizing-type OSCCs and 27 of 90 OSCC tissues [59,62]. Nomura et al. have suggested that ATG16L1 abundant stromal expression was associated with lympho-vascular invasive tumor cells development and positive lymph node status [60].

ATG5 is covalently bound to ATG12. Along with ATG16L1, ATG5 is mainly involved in the phagophore elongation [62]. Dual expression of ATG5 and BECN1 should be regarded as a bad prognostic indicator for OSCC diagnosed patients [55,63].

SQSTM1 is a receptor protein mainly involved in the coordination of selective autophagy and ubiquitination [64]. Liu et al. research revealed that the increased LC3-II expression enhanced SQSTM1 cytoplasmic level. Liu et al. also have shown that excessive SQSTM1 was associated with aggressive clinicopathological features and bad prognosis [59,65]. Moreover, it seemed that excessive SQSTM1 could contribute to glutathione induction, triggering resistance to cytotoxic radiation [66].

BECN1 represents an essential modulator of phagophore nucleation and, also, an important player in tumor suppression molecular mechanism [67]. Specific allelic deletions of BECN1 gene have been found in most human breast, ovarian and prostate cancers [68]. BECN1 and LC3 are two critical players in autophagy induction. Wang Y et al. results revealed reduced levels of BECN1 and LC3 in tongue squamous cell carcinoma tissues and squamous cell carcinoma lines [50,69]. Kapoor et al. also observed low expression of BECN1 mRNA and reduced BECN1 protein levels in other OSCC tissues [56,70]. Wang’s group have also shown that reduced BECN1 results in decreased ATG4, ATG5 and LC3-II levels, as well as intensified proliferation, migration and invasion of tongue SCC cells [50,69]. On the contrary, overexpression of BECN1 exerts converse effects [50,69,71]. Autophagy can be considered an important actor in both the pathogenesis and treatment response in oral cancer. Jiang et al. revealed that autophagy could have a significant impact on tumorigenesis and tumor progression in primary salivary gland adenoid cystic carcinoma (ACC) [72]. Liang et al. experimental results revealed a significant correlation between BECN1 and unfavorable prognosis in ACC [73].

#### 3.2.1. Oncogenes and Tumor Suppressors that Control the Autophagy Pathway

It is very important to notice that many autophagy-inducing proteins are either tumor suppressor proteins or oncoproteins (Table 1) [74]. More specifically, it has been highlighted that tumor suppressors that negatively regulate mTOR, (PTEN, AMPK, LKB1, and TSC1/2) initiate autophagy while mTOR activators (such as AKT, class I PI3K, Ras, inhibit autophagy, suggesting that autophagy may have a crucial role in tumor evolution [74].

(1) **mTOR protein kinase** represents the major negative regulator of autophagy [74]. This kinase is involved in many signaling pathways controlling cell growth, mainly downstream of growth factor receptors with tyrosine kinase activity. Constitutive activation of these receptors, activating mutations of Ras, PI3K, AKT and the inactivating mutations of negative regulators, such as PTEN, are all frequently met during cancer development, suggesting that inhibition of autophagy likely contributes to the onset of tumor progression [74]. Martins et al. immunohistochemical investigation in oral epithelial dysplasia revealed a greater expression of AKT and mTOR activated forms, compared to OSCC and non-dysplastic oral tissues [75]. Moreover, mTOR immunohistochemical analysis in both HPV (-) and HPV-associated HNSCC lesions have highlighted its important role as a molecular target in oral cancer [76]. Harsha et al. study revealed a higher expression of AKT and mTOR in human ameloblastoma tissues compared to normal oral mucosa [76]. Matsuo et al. hypothesized that the downstream protein of AKT/mTOR pathway, GSK3, represents one of the first steps in cervical lymph node metastasis in the OSCC context [77]. Harsha et al. have outlined AKT/mTOR signaling pathway’s role in the initiation, development and progression of oral verrucous carcinoma [76]. Ferreira et al. have studied the level of expression of several regulatory proteins in OSCC cells. Their study revealed high levels of AKT and mTOR active forms in OSCC tissues from alveolar ridge, gingiva and hard palate and, leading to the conclusion that AKT/mTOR pathway’s activation should be associated with OSCC development [78].

All these studies outlined the AKT/mTOR pathway significance in the molecular landscape of oral cancer initiation and progression. The upregulation or overexpression of this pathway trigger tumor growth and cause poor prognosis, especially by the way they influence autophagy [79]. It has been pointed out that the neutrophil gelatinase-associated lipocalin (NGAL) knockdown induced mTOR activation and, consequently, suppressed autophagy, thereby sustaining oral cancer progression. This study has also revealed the involvement of the AKT/mTOR pathway in NGAL-mediated control of autophagy in oral cancer cells [80].

(2) **PTEN (phosphatase and tensin homolog deleted on chromosome 10)** is regarded as the “new guardian of the genome”. On the one hand, PTEN plays a significant role in the molecular landscape of cell survival and proliferation. On the other hand, it is deeply involved in the differentiation and apoptosis pathways. PTEN ranks in second place regarding mutations frequency in cancer, after P53 [81].

Several studies have examined the relationship between PTEN and autophagy in many different model systems. For instance, De Amicis et al. reported that in breast cancer cells, progesterone triggered, via its receptor - PR-B, PTEN activation. Moreover, activated PTEN has been shown to induce the downregulation of the PI3K/AKT pathway, consequently stimulating autophagy, which, in turn, led to reduced cell survival [81]. De Amicis et al. have also demonstrated that 5-methoxypsoralen treatment of breast cancer cell lines has induced autophagy by positively regulating Beclin-1, PI3K-III, UVRAG expression and by LC3-I to LC3-II conversion [81]. In conclusion, De Amicis et al. study highlighted PTEN’s concrete involvement in the autophagy induction [81].

Downregulation of PTEN has been also reported in the oral cancer context, possibly being caused by epigenetic modifications, mostly hypermethylation. Kurasawa et al. represent one of the research groups that support this molecular mechanism regarding PTEN regulation in OSCC context [46].

The precise molecular mechanisms behind PTEN involvement in the oral cancer molecular landscape are still incompletely elucidated. However, it is very likely that autophagy represents one of PTEN’s main targets to regulate, in oral cancer as well.

(3) **Beclin-1**, one of the most important autophagy regulators, functions as a tumor suppressor in mammalian cells. Beclin-1 is included in the class III PI3K complex that promotes autophagy. It is very important to notice that the monoallelic mutations of *Beclin-1* gene have been frequently reported in prostate, ovarian, and breast cancers in humans. These experimental observations outlined the *Beclin-1* role as a haplo-insufficient tumor suppressor involved in the molecular mechanisms of several human cancers [74].

(4) **The death-associated protein kinase, DAPK**, a kinase that phosphorylates Beclin-1, disrupting the Beclin-1/BCL-2 complex. It has been revealed that DAPK gene, an autophagy inducer, is frequently silenced by methylation in different types of human cancers [82].

(5) **BCL-2 and BCL-XL**, important players in the inhibition of apoptosis, have also been shown to be involved in oncogenesis, as autophagy negative regulators. Although BCL-2 and BCL-XL are not directly involved in mTOR signaling, they can interact with the Beclin-1 BH3 domain and sequester Beclin-1 as an inactive complex in the ER [74].

(6) Recently has been brought to light an important autophagy negative regulator—the **protein c-FLIP** (**cellular FLICE-like inhibitory protein**) [83]. c-FLIP is, also, an apoptosis-inhibitor of the extrinsic apoptotic pathway by suppressing death receptor-induced caspase 8 activation [83]. Lee et al. study on T lymphocytes revealed that c-FLIP has an important role in both, autophagy and apoptosis regulation. c-FLIP’s mission is to prevent Atg3 binding to LC3, consequently, negatively regulating the autophagosome assembly. [84].

(7) A special role is assigned to the **P53 protein**. Considered a true genome guardian, P53 plays a crucial role in the DNA repair mechanism, cell cycle control, cellular differentiation and apoptosis [85]. Sasahira et al. highlighted that P53 somatic mutations have been detected in 10% of oral dysplasia and in 60–80% of OSCC [85]. Furthermore, the GenomeWide Association Study revealed the usual presence of mutated P53 in the cases of human papillomavirus-negative OSCC [85]. It also has been pointed that the overall survival of P53-mutant OSCC patients was much worse, compared with that of OSCC patients with the wild-type P53 [85].

Oikawa et al. study, using a next-generation sequencing in OSCC tissues, revealed that P53, CDKN2A, PIK3CA mutations combined with PIK3CA and AKT1 copy number amplification, triggered distant metastasis and, consequently, a significantly poorer prognosis in the studied group [86].

Recent experimental evidence rigorously sustain that P53 should be regarded either as an inhibitor or an activator of autophagy, depending on its subcellular localization and its downstream signal pathway. This finding gains particular significance as P53 deficiency or mutant variants of P53 that accumulate in the cytoplasm of tumor cells enable activation of autophagy [87].

P53 has the ability to co-regulate autophagy and apoptosis. P53 controls autophagy-related pathways, AMPK/mTOR and Bmf/Beclin-1, and, also, modulates the expression of apoptosis-related genes, Bcl-2 and Apaf1 [88]. Autophagy and apoptosis are strange partners influencing each other. Autophagy and apoptosis cross-talk represents a crucial molecular event to the cell fate. However, their molecular relationship is quite complicated by their contradictory roles under some circumstances.

#### 3.2.2. Autophagy Regarded as a Tumor Suppressor

The first important experimental data sustaining the possible tumor suppressor role of autophagy were obtained in studies targeting Beclin-1. *Beclin-1* gene monoallelic loss on chromosome 17q21, has been reported in 40–75% of human ovary, breast and prostate tumors, suggesting that autophagy may play the role of a tumor suppressor [89]. Furthermore, Beclin-1^+/−^ mice have shown a high incidence of spontaneous tumors, especially lymphoma and hepatocellular carcinoma. Consequently, the experimental evidence presented suggests that beclin-1 functions as a haplo-insufficient tumor suppressor gene. Wei et al. have shown that the EGFR-dependent Beclin-1 phosphorylation on several tyrosine residues, decreased the activity of the Beclin-1/PI3KC3 complex and, consequently, inhibited autophagy in non-small-cell lung carcinoma cells. This effect was reduced in the presence of an EGFR kinase inhibitor. [67,90].

Concerning oral cancer, interesting experimental evidence sustained that activated autophagy was able to induce oral cancer cells survival decline [91,92,93]. In this context, survivin, a usually expressed protein in head and neck squamous cell carcinoma (HNSCC) patients, should be mentioned. This protein has been associated with poor survival and chemotherapy resistance in HNSC. Zhang et al. study revealed that survivin overexpression was negatively correlated with the autophagic marker LC3, in human HNSCC cells [91].

Han et al. results suggested that sulfasalazine promoted autophagic cell death via Akt and ERK pathways, having chemotherapeutic potential for the oral cancer treatment [93].

Taken together, the evidence presented above contribute in supporting the hypothesis that autophagy should be regarded as a one of the main actors in tumor suppression, at least in the early stages of oral cancer. However, these evidences also highlight the dual nature of autophagy during tumor development and progression.

The possible molecular strategies that sustain the tumor suppressor role of autophagy:

##### Autophagic Cell Death

Autophagy represents primarily a mechanism that insures cell survival under stress conditions. However, there is evidence indicating that, under specific conditions, an increase of the autophagic flux may induce cell death, explaining the possible tumor suppressor effects of this Janus God like molecular pathway (Table 2) [94]. Pattingre et al. have shown that the expression of a mutant Beclin-1, unable to interact with BCL-2, induced autophagy to a greater extent compared to the wild-type Beclin-1, triggering cell death (Table 2) [95]. Zhao et al. highlighted that the transcription factor FoxO1 have induced autophagy in a manner independent of its transcriptional activity, triggering autophagic cell death in tumor cells. These results suggest that the cytosolic FoxO1 promoted autophagy acted as a tumor suppressor mechanism (Table 2) [45].

##### Autophagic Senescence

A controversial strategy that may sustain the autophagy’s tumor suppressor activity, is its role played in the senescence molecular cascade. Young et al. (Table 2) [96] showed that in fibroblasts autophagy is activated during senescence that has been induced by the oncogene Ras. In this context, autophagy inhibition delayed but did not block the deployment of the oncogene-mediated senescence. These data are important because senescence should be regarded as a major intrinsic barrier against cell malignant transformation, although this barrier protection may be only temporary (Table 2) [97].

##### Inflammation

The tumor microenvironment is characterized by complex molecular interactions between different cell types coexisting within tumor. The crosstalk between these cells control and regulates tumor progression. In this context, it is important to note that both inflammatory cells and cytokines are main actors because a proinflammatory environment can induce malignant cells survival and proliferation, stimulates angiogenesis, metastasis, and control the response to chemotherapy (Table 2) [98]. Degenhardt et al. have shown that autophagy inhibition in apoptosis-deficient tumor cells promoted local inflammatory reactions and tumor growth (Table 2) [99]. These results led to the hypothesis that autophagy may act as a tumor suppressor by reducing the intensity level of local inflammatory reactions. The anti-inflammatory effect of autophagy has been suggested to be sustained by the removal of cell and corpses (Table 2) [74,100]. Moreover, a complex connection between the immune response and the autophagy has been highlighted, outlining autophagy’s role as a subtle but efficient tumor suppressor (Table 2) [101]. For instance, the LC3-conjugation system (LC3, ATG4A–D, ATG7, ATG3), important for isolation membrane elongation and/or complete closure, inhibits type I IFN production [102] and pro-inflammatory cytokine production(Table 2) [103], maintains T cells number [7,104] and is involved in the intestinal immune epithelial cell function (Table 2) [105].

##### Oxidative Stress and Genomic Instability

One of the most challenging and subtle strategy of autophagy as a tumor suppression is via the regulation of cellular redox homeostasis, by controlling reactive oxygen species (ROS) production. Increased ROS production can induce mutagenesis, upregulating the oncogenes activation and, consequently, initiate carcinogenesis (Table 2) [106]. Mitochondria is regarded as the main source of intracellular ROS. Mitochondrial ROS production increases as these organelles become damaged or age (Table 2) [107]. In this context, autophagy intervenes by selectively degrading the damaged mitochondria, a molecular mechanism known as mitophagy. Consequently, autophagy inhibition will trigger genotoxic effects, genomic instability and oncogenes activation of oncogenes by increased ROS production (Table 2) [108], molecular events reported in autophagy-defective cells [108]. Thus, potentially damaged mitochondria selective removal (mitophagy) reduces excessive ROS production and thereby limits tumor-promoting effects dependent on the production of such species (Table 2) [109].

Moreover, autophagy also responsible for the protein aggregates degradation. Disruptions in the autophagic pathway have been correlated with the accumulation of the autophagy substrate P62 and protein aggregates. These molecular events are considered to induce increased ROS production, ER oxidative stress, and, consequently, activation of the DNA damage response [108]. The selective autophagy substrate, P62, that accumulates when autophagy intensity is reduced, contains: an UBA domain—or binding to polyubiquitinated proteins; a PB1 domain—responsible for protein oligomerization and an LIR domain (LC3-interacting region)—for association with LC3. Due to its structural characteristics, P62 insures selective degradation of both polyubiquitinated proteins and organelles, such as mitochondria (Table 2) [109,110]. Interestingly, Lau et al. reported increased P62 levels in human tumors. Moreover, P62 accumulation induce NRF-2-dependent antioxidant defense upregulation, which, in turn, may contribute to tumor progression (Table 2) [111].

#### 3.2.3. Autophagy Regarded as a Tumor Growth Promoter

The tumor growth promotor face of autophagy is based on the tumoral cells need to adapt to ischemia in a hypoxic and nutrient deprived environment. According to these, autophagy becomes activated in the hypoxic regions of tumors. Degenhardt et al. have reported that autophagy inhibition by monoallelic deletion of *beclin-1* (*Bcn1* ^+/−^) induced cell death, specifically in those regions. These findings outlined the autophagy’s role as tumor cells’ survival promotor, under conditions of metabolic stress (Table 3) [99]. Furthermore, the tumor cells high proliferation rates impose higher biosynthetic, and consequently, bioenergetic needs, compared to non-malignant cells. These elevated requirements can be sustained by inducing autophagy, as a mechanism that will insure both ATP and metabolic intermediates production (Table 3) [100].

Guo et al. highlighted that in the case of activated Ras oncogene tumor cells, survival is insured by high levels of basal autophagy. These tumor cells become vitally dependent on the autophagy pathway (Table 3) [88]. These findings led to the conclusion that autophagy is able to promote tumor cell survival by increasing the stress tolerance and providing a pathway that insures necessary nutrients in order to support the enhanced energetic requirements of these cells [100].

In the context of activated Ras- driven cancers, Mathew et al. presented autophagy as a mechanism that ensures an adequate mitochondrial metabolism by supplying mitochondrial intermediates, obtained by macromolecules degradation, in both starvation and basal conditions (Table 3) [108]. In conclusion, it can be said that particularly, activated Ras-dependent tumorigenesis seemed to be actually “addicted to autophagy” in order to obtain energetic and metabolic support for rapid tumor growth.

Huo et al. have shown that autophagy partial inhibition by monoallelic loss of Beclin-1 (*Bcn1* ^+/−^) also stimulated apoptosis and significantly slowed down tumor growth via P53 activation. Consequently, the authors proposed that autophagy is able to promote tumor growth by P53 suppression when DNA has been damaged (Table 3) [112]. These findings outlined the idea that autophagy can display its face as a tumor progression promoter, also, in a manner independent of activated Ras [112].

**Figure 2:**
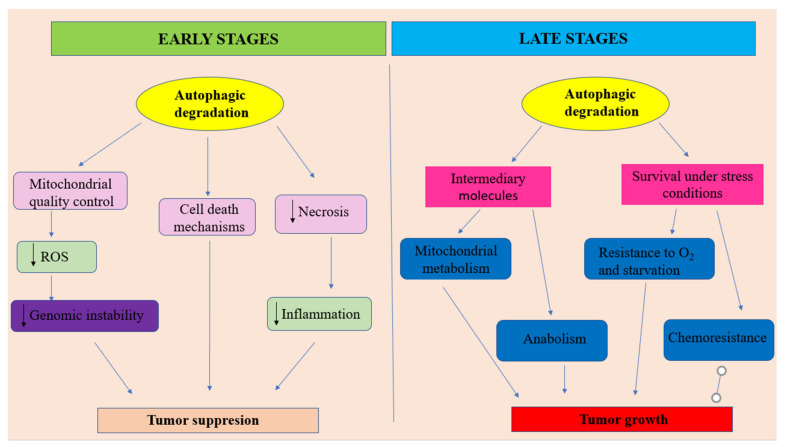
The dual character of autophagy in oral cancer.

Analyzing all that has been presented above, it can be outlined that autophagy should be regarded as a double—faced molecular Janus god. On the one hand, at early stages of tumor development, autophagy is able to act as a tumor suppressor by increasing the damaged proteins and organelles (mostly mitochondria) degradation (Figure 2). In this role, autophagy acts as an efficient regulatory system that controls ROS production, insuring genomic stability. Moreover, autophagy is able to prevent necrotic cell death in apoptosis-defective cells, decreasing in this way the local inflammatory reactions’ intensity and, consequently, reducing tumor development. Additionally, sometimes, autophagy may direct the cellular molecular events towards autophagic cell death. On the other hand, especially, at later stages of tumor evolution, under metabolic stress conditions, activated autophagy provides tumor cells nutrients for energy production and metabolic intermediates for biosynthetic pathways, in order to sustain tumoral cells survival and tumor growth. In this context, autophagy, also acts as a promotor of the resistance to cancer therapy (Figure 2).

- at early stages of tumor development, autophagy plays the role of a tumor suppressor by ensuring damaged proteins and organelles degradation. In this context, autophagy should be regarded as controlling system, able to decreases ROS production and, consequently, maintaining genomic stability. Autophagy also can prevent necrotic cell death in apoptosis-defective cells, ensuring in this way the decrease of local inflammation and tumor growth. In some situations autophagy can lead to apoptotic cell death.
- at later stages of tumor evolution, activated autophagy plays the role of cancer cell survival and tumor growth promoter, by suppling metabolic stressed tumor cells with nutrients, in order to sustain energy generation in mitochondria and biosynthetic pathways. Unfortunately, autophagy represents one of the main actors in developing the resistance to cancer therapy. Adapted from [100].

#### 3.2.4. Autophagy Related Chemoresistance in Oral Cancer

Due to its dual capacity to either induce cell death or promote cell survival, important evidence pointed that autophagy has a two-faced role in response to chemotherapy in cancer. Important experimental evidence has sustained autophagy’s potential as a therapeutic target for oral cancer [113,114,115].

On the one hand, autophagy inhibition can enhance the cisplatin sensitivity in OSCC, hypopharyngeal carcinoma and salivary adenoid cystic carcinoma [114,116,117,118].

On the other hand, DNA-damaging agents (cisplatin, methotrexate and 5-fluorouracil) are able to induce autophagy with a cytoprotective effect [119,120]. Beclin-1, Atg12-Atg5 and LC3-II enhanced expression together with the autophagosome formation were observed in the methotrexate-resistant SCC-9 cell line compared with the sensitive SCC-9 cell line [121]. Similar results were reported in a laryngeal cancer study, in which exposure to cisplatin induced autophagosomes aggregation in the cytoplasm and enhanced Beclin-1 and LC3II expression [122]. Consequently, the induction of autophagy has attenuated the cisplatin treatment cytotoxicity expression [122]. All these results, taken together, outline the conclusion that, at a certain time, autophagy enhancement may play a key role in the chemoresistance mechanism in head and neck cancers.

Autophagy inhibition should be regarded as a potential target in order to reverse chemoresistance in cancer treatment. However, it must not be forgotten that autophagic cell death could also be induced in oral cancer cells in order to induce tumor cell death. Therapeutic molecules like sulfasalazine thymoquinone and tetrandrine were also shown to have anticancer effects by inducing autophagic cell death. These results highlight the idea that autophagic cell death induction should also be regarded as an alternative approach to destroy tumor cells [76,93,123,124,125,126,127].

In conclusion, understanding how to overcome cytoprotective autophagy and how to take advantage of autophagic cell death is critical in order to enhance the cancer cells’ sensitivity to particular therapeutic agents.

## 4. Conclusions

The important progress made in the molecular landscape of autophagy opened new insights into the pathogenesis of oral cancer.

More and more experimental data emphasizes the duality of autophagy, a tumor suppressor, especially at early stages of tumor development and a tumor promoter, at later stages of tumor evolution. However the exact reason and moment of autophagy’s role change, are still unknown.

Also, further studies are imposed in order to better understand the complex molecular interactions between autophagy, immune response, immune response and apoptosis, in the oral cancer context. It can be anticipated that future, more detailed incursions into the autophagy landscape may lead to novel targets’ identification, so necessary for elaborating new and efficient therapeutic strategies.

## Figures and Tables

**Table 1:** List of oncogene products and tumor suppressors that control the autophagy pathway.

Oncogene Product or Tumor Suppressor	Effect on Autophagy Pathway	Reference
(1) mTOR	Negative regulator	[74,75,76,77,78,79,80]
(2) PTEN	Inducer	[46,81]
(3) Beclin-1	Inducer	[74]
(4) DAPK	Inducer	[82]
(5) BCL-2; BCL-XL	Negative regulator	[74]
(6) c-FLIP	Negative regulator	[83,84]
(7) P53	Negative regulator/Inducer	[85,86,87,88]

**Table 2:** Summary of analyzed publications highlighting the tumor suppressor role of autophagy.

Publication Title	Proposed Molecular Mechanism for Sustaining the Tumor Suppressor Role of Autophagy	Reference
Autophagic cell death: the story of a misnomer	**Autophagic cell death**	[94]
Bcl-2 antiapoptotic proteins inhibit Beclin 1-dependent autophagy	**Autophagic cell death**	[95]
Anti-neoplastic activity of the cytosolic FoxO1 results from autophagic cell death	**Autophagic cell death**	[45]
Autophagy mediates the mitotic senescence transition	**Autophagic senescence**	[96]
The dynamic nature of autophagy in cancer	**Autophagic senescence**	[97]
Cancer-related inflammation	**Inflammation downregulation**	[98]
Autophagy promotes tumor cell survival and restricts necrosis, inflammation, and tumorigenesis	**Inflammation downregulation**	[99]
The double-edged sword of autophagy modulation in cancer	**Inflammation downregulation**	[74]
The Roles of Autophagy in Cancer.	**Inflammation downregulation**	[100]
Autophagy in immunity and inflammation	**Inflammation downregulation**	[101]
The Atg5–Atg12 conjugate associates with innate antiviral immune responses.	**Inflammation downregulation**	[102]
Loss of the autophagy protein Atg16L1 enhances endotoxin-induced IL-1β production	**Inflammation downregulation**	[103]
Autophagy in mammalian development and differentiation	**Inflammation downregulation**	[7]
Autophagy in health and disease: A comprehensive review.	**Inflammation downregulation**	[104]
Virus-plus-susceptibility gene interaction determines Crohn’s disease gene *Atg16L1* phenotypes in intestine	**Inflammation downregulation**	[105]
Reactive species: a cell damaging rout assisting to chemical carcinogens	**Oxidative stress and genome instability**	[106]
Mitochondrial gateways to cancer	**Oxidative stress and genome instability**	[107]
Autophagy suppresses tumor progression by limiting chromosomal instability	**Oxidative stress and genome instability**	[108]
Oncosuppressive functions of autophagy	**Oxidative stress and genome instability**	[109]
PINK1/Parkin-mediated mitophagy is dependent on VDAC1 and p62/SQSTM1	**Oxidative stress and genome instability**	[110]
A noncanonical mechanism of Nrf2 activation by autophagy deficiency: direct interaction between Keap1 and p62	**Oxidative stress and genome instability**	[111]

**Table 3:** Summary of analyzed publications highlighting the tumor growth promoter role of autophagy.

Publication Title	Proposed Molecular Mechanism	Reference
Autophagy promotes tumor cell survival and restricts necrosis, inflammation, and tumorigenesis	**Beclin-1 dependent regulation**	[99]
The double-edged sword of autophagy modulation in cancer.	**Stress tolerance increase**	[74]
The Roles of Autophagy in Cancer.	**Inflammation downregulation**	[100]
Targeting GRP75 improves HSP90 inhibitor efficacy by enhancing P53-mediated apoptosis in hepatocellular carcinoma	**Ras activation**	[88]
Autophagy suppresses tumor progression by limiting chromosomal instability	**Ras activation**	[108]
Autophagy opposes P53-mediated tumor barrier to facilitate tumorigenesis in a model of PALB2-associated hereditary breast cancer	**P53 suppression**	[112]
